# Supplementary material for: Two different and robustly modeled DNA binding modes of Competence Protein ComP - systematic modeling with AlphaFold 3, RoseTTAFold2NA, Chai-1 and re-docking in HADDOCK
Source: PLoS One. 2025 May 8;20(5):e0315160. doi: 10.1371/journal.pone.0315160 (PMC12061091; doi:10.1371/journal.pone.0315160)
Supplement: S1 Table — (PDF) [file pone.0315160.s001.pdf]

Table S1 The seven different ComP investigated with their accession numbers, primary structure and paired DUS-dialect.

Species	GenBank/RefSeq accession	ComP primary structure (N-terminally truncated)	DUS-dialect 5'-3'	DUS-dialect name
Neisseria subflava NJ9703	EFC51080.1	RSANLRAAHAALLENARFMEQFYAKKGSFKLTSTKWPELPVKEAGGFCIRMSGQAKGILEGKFTLKAVALDREAEPRVLRLNESLTAVVCGKMKGKGSCTDGEEIFRGNDAECRPFTG	AGGCCGTCTGAA	AG-DUS
Bergeriella denitrificans NCTC10295	STZ76941.1	REGRLREAQAALLENAQFLEKHYRQTGSIRANSTTWPTLPVTEAGGFCIRLSGLARGQSNQTEGKFTLKAVALDKTREPRVLKTNEALMTTICESSSSSCDDGLQHFSGDGSTDQDCRVYQH	AGGCCGTCTGAA	AG-DUS
Neisseria mucosa ATCC 25996	EFC87706.1	RDSEMRQALAALVESAQFMERFYQQNGSFKKTSTAWPDLPNSRSLENFCIYPHGLARGALDGKFTLKAVALDKNKEPRVIKINESLTTFICESTASSCDDVTKNYFSGADKNCSVYRL	AGGCCGTCTGAA	AG-mucDUS
Eikenella corrodens ATCC 23834	EEG23425.1	RKSRLEEANAALLENSRAMERFYARNRTFKATSTTWPALAVSQTQHFCIKFQGNARGVLGDKYTIKAVAFDVSKEPRVLLINQDQTVRICQSSRSRCDNKEVFSGGNNIDQECELLH	AGGCTACCTGAA	AG-eikDUS
Kingella denitrificans ATCC 33394	EGC16987.1	RKSRLSEVQQLMLDNAQAWERHYAAHGHYRQTSRKWAALPVQGNDDFCIRPQGAPRGAAHDGQYSLKAVALDKTKEPRVLVMNQDLTFLLCEESSSTCAETDYFANPARADKNCRSYP	AAGCAGCCTGCA	AA-king3DUS
Neisseria meningitidis MC58	WP_002214937.1	EKAKINAVRAALLENAHFMEKFYLQNGRFKQTSTKWPSLPIKEAEGFCIRLNGIARGALDSKFMLKAVAIDKDKNPFIIKMNENLVTFICKKSASSCSDGLDYFKGNDKDCKLLK	ATGCCGTCTGAA	AT-DUS
Neisseria cinerea ATCC	WP_003678313.1	EKARISAVRSALLENAHFMEKFYLQNGTFKQTSTKWPKLPIQEAEGFCIR	ATGCCGTCTGAA	AT-DUS

14685		LNGVARGALDSKFMLKAVAIDKNK EPRIIKMNENLVTFVCKGSTSSCDD GLDYFRGNDKGCTLFK		
