# Supplementary material for: Two different and robustly modeled DNA binding modes of Competence Protein ComP - systematic modeling with AlphaFold 3, RoseTTAFold2NA, Chai-1 and re-docking in HADDOCK
Source: PLoS One. 2025 May 8;20(5):e0315160. doi: 10.1371/journal.pone.0315160 (PMC12061091; doi:10.1371/journal.pone.0315160)
Supplement: S2 Table — (PDF) [file pone.0315160.s002.pdf]

Table S2 The different MSA schemes used in Chai-1, list of the applied settings.

MSA scheme	MSA for ComP	MSA for DUS	Origin of MSA for ComP	Origin of MSA for DUS	Paired MSA	ComP MSA query coverage
1	Yes	Yes	BFD	Genomic DUS counts	Yes	50.00%
2	Yes	Yes	Uniref30	Genomic DUS counts	Yes	50.00%
3	Yes	Yes	BFD	Genomic DUS counts	Yes	75.00%
4	Yes	Yes	Uniref30	Genomic DUS counts	Yes	75.00%
5	Yes	Yes	BFD-Uniref30 composite	Genomic DUS counts	Yes	75.00%
6	Yes	No	BFD	NA	No	50.00%
7	Yes	No	Uniref30	NA	No	50.00%
8	Yes	No	BFD	NA	No	75.00%
9	Yes	No	Uniref30	NA	No	75.00%
10	Yes	No	BFD-Uniref30 composite	NA	No	75.00%
