# Supplementary figures and images for: Two different and robustly modeled DNA binding modes of Competence Protein ComP - systematic modeling with AlphaFold 3, RoseTTAFold2NA, Chai-1 and re-docking in HADDOCK
Source: PLoS One. 2025 May 8;20(5):e0315160. doi: 10.1371/journal.pone.0315160 (PMC12061091; doi:10.1371/journal.pone.0315160)

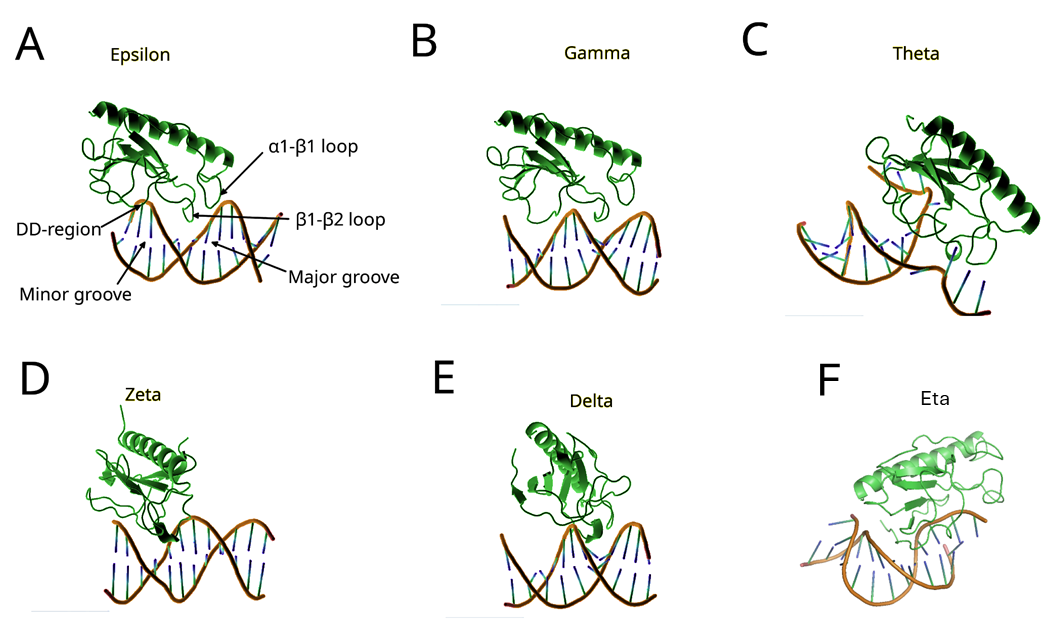

Supplement: S1 Fig — Epsilon, B. Gamma, C. Theta, D. Zeta, E. Delta and F. Eta modeled in AF3, Chai-1 and RF2NA. (TIF) [file pone.0315160.s003.tif]

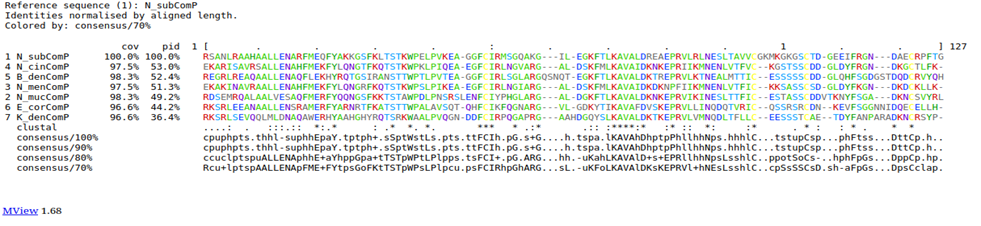

Supplement: S2 Fig — The first 28 amino acids of the N. meningitidis mature ComP are trimmed away, as are the other ComPs accordingly. (TIF) [file pone.0315160.s004.tif]

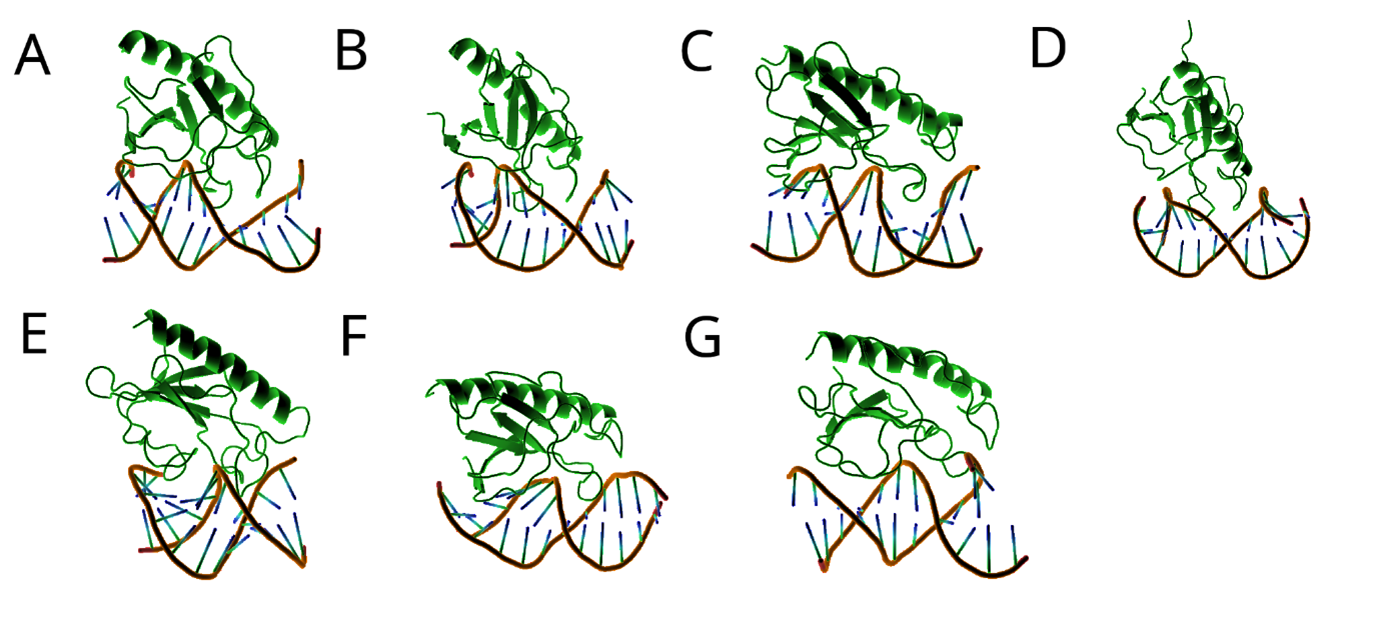

Supplement: S3 Fig — A. N. mucnat (Epsilon). B. N. mennat (Epsilon). C. K. dennat (Epsilon). D. E. cornat (Epsilon). E. B. dennat (Epsilon). F. N. cinnat (Gamma). G. N. subnat (Gamma). AF3, Chai-1 and RF2NA DNA binding modes were used to guide the assignment of grooves in the HADDOCK distorted DNAs. (TIF) [file pone.0315160.s005.tif]
