# Supplementary material for: Two different and robustly modeled DNA binding modes of Competence Protein ComP - systematic modeling with AlphaFold 3, RoseTTAFold2NA, Chai-1 and re-docking in HADDOCK
Source: PLoS One. 2025 May 8;20(5):e0315160. doi: 10.1371/journal.pone.0315160 (PMC12061091; doi:10.1371/journal.pone.0315160)

Figure S7. Representative PAE plots of the top ranking scrambled AF3 model for each ComP$_{scr}$ and assigned mode.

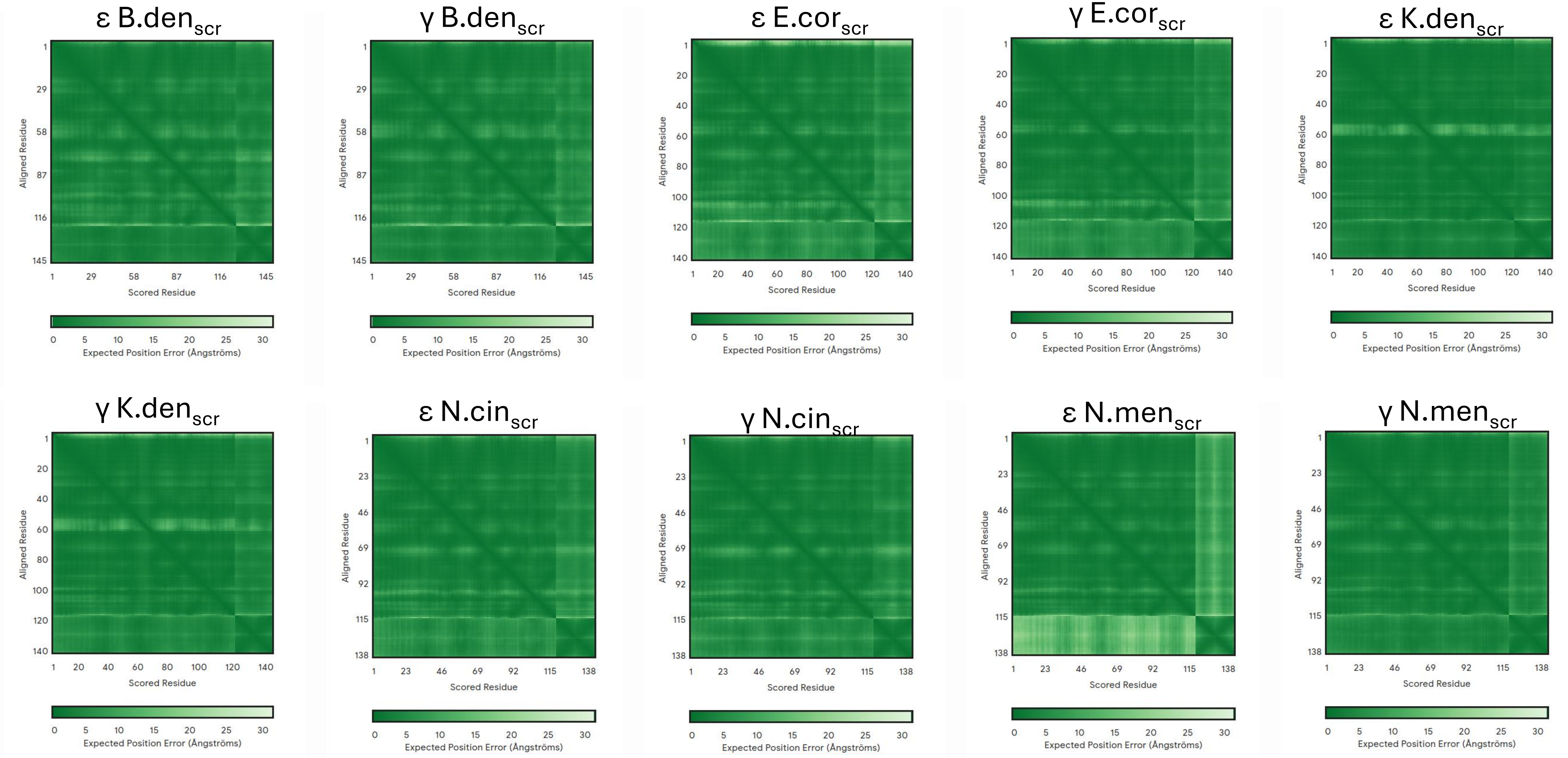

Figure S7. continued

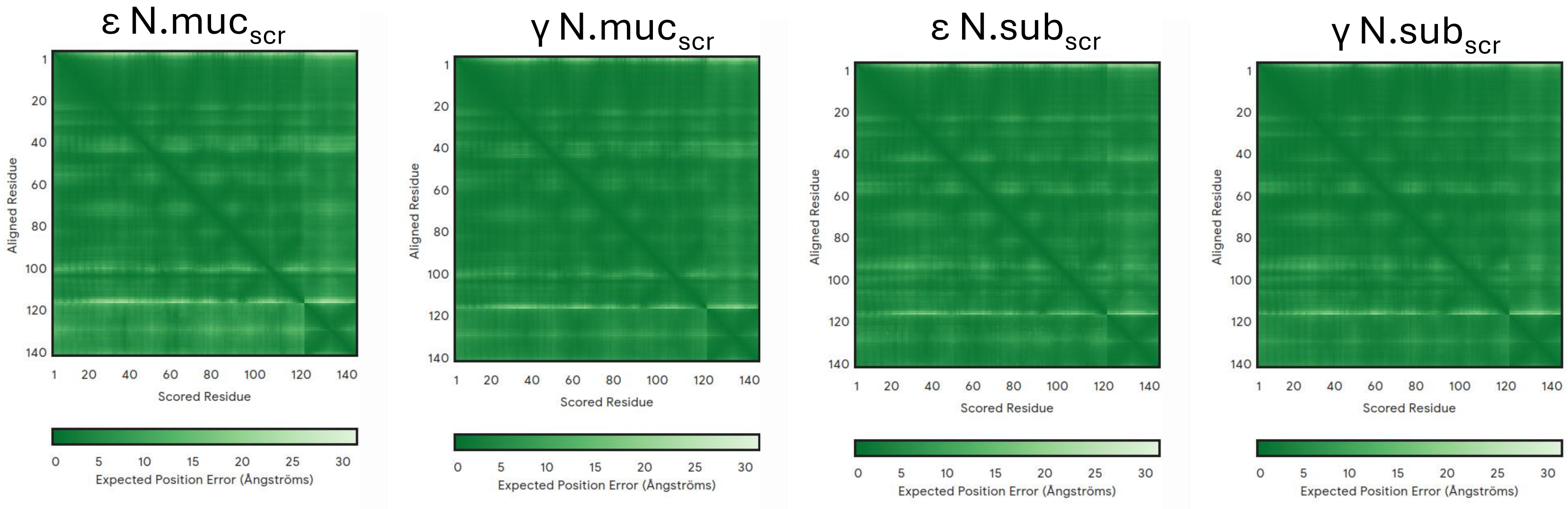

Supplement: S7 Fig — (PDF) [file pone.0315160.s009.pdf]
