# Supplementary material for: Two different and robustly modeled DNA binding modes of Competence Protein ComP - systematic modeling with AlphaFold 3, RoseTTAFold2NA, Chai-1 and re-docking in HADDOCK
Source: PLoS One. 2025 May 8;20(5):e0315160. doi: 10.1371/journal.pone.0315160 (PMC12061091; doi:10.1371/journal.pone.0315160)
Supplement: S1 Code Output — Median PAE and pLDDT values are also shown. (PDF) [file pone.0315160.s010.pdf]

Code Output S1. Wilcoxon rank-sum tests on PAE and pLDDT, comparing AF3, Chai-1 and RF2NA. Median PAE and pLDDT values are also shown.

Species: **B_denitrificans**
Pairwise Wilcoxon test for PAE:

Pairwise comparisons using Wilcoxon rank sum test with continuity correction

data: PAE and Software

	AF3	Chai-1
Chai-1	0.019	-
RF2NA	8.3e-14	1.2e-12

P value adjustment method: bonferroni
Pairwise Wilcoxon test for pLDDT:

Pairwise comparisons using Wilcoxon rank sum test with continuity correction

data: plddt and Software

	AF3	Chai-1
Chai-1	0.92	-
RF2NA	4.2e-13	1.2e-12

P value adjustment method: bonferroni

Species**: E_corrodens**
Pairwise Wilcoxon test for PAE:

Pairwise comparisons using Wilcoxon rank sum test with continuity correction

data: PAE and Software

	AF3	Chai-1
Chai-1	<2e-16	-
RF2NA	<2e-16	<2e-16

P value adjustment method: bonferroni
Pairwise Wilcoxon test for pLDDT:

Pairwise comparisons using Wilcoxon rank sum test with continuity correction

data: plddt and Software

	AF3	Chai-1
Chai-1	<2e-16	-
RF2NA	<2e-16	<2e-16

P value adjustment method: bonferroni

Species: **K_denitrificans**
Pairwise Wilcoxon test for PAE:

Pairwise comparisons using Wilcoxon rank sum test with continuity correction

data: PAE and Software

	AF3	Chai-1
Chai-1	1	-
RF2NA	<2e-16	<2e-16

P value adjustment method: bonferroni
Pairwise Wilcoxon test for pLDDT:

Pairwise comparisons using Wilcoxon rank sum test with continuity correction

data: plddt and Software

	AF3	Chai-1
Chai-1	<2e-16	-

RF2NA	<2e-16	<2e-16

P value adjustment method: bonferroni

Species**: N_cinerea**
Pairwise Wilcoxon test for PAE:

Pairwise comparisons using Wilcoxon rank sum test with continuity correction

data: PAE and Software

	AF3	Chai-1
Chai-1	<2e-16	-
RF2NA	<2e-16	<2e-16

P value adjustment method: bonferroni
Pairwise Wilcoxon test for pLDDT:

Pairwise comparisons using Wilcoxon rank sum test with continuity correction

data: plddt and Software

	AF3	Chai-1
Chai-1	< 2e-16	-
RF2NA	1.8e-11	< 2e-16

P value adjustment method: bonferroni

Species: **N_meningitidis**
Pairwise Wilcoxon test for PAE:

Pairwise comparisons using Wilcoxon rank sum test with continuity correction

data: PAE and Software

	AF3	Chai-1
Chai-1	1.5e-08	-

RF2NA	< 2e-16	1.9e-08

P value adjustment method: bonferroni
Pairwise Wilcoxon test for pLDDT:

Pairwise comparisons using Wilcoxon rank sum test with continuity correction

data: plddt and Software

	AF3	Chai-1
Chai-1	1.5e-08	-
RF2NA	< 2e-16	1.8e-08

P value adjustment method: bonferroni

Species**: N_mucosa**
Pairwise Wilcoxon test for PAE:

Pairwise comparisons using Wilcoxon rank sum test with continuity correction

data: PAE and Software

	AF3	Chai-1
Chai-1	< 2e-16	-
RF2NA	1.7e-07	4.1e-08

P value adjustment method: bonferroni
Pairwise Wilcoxon test for pLDDT:

Pairwise comparisons using Wilcoxon rank sum test with continuity correction

data: plddt and Software

	AF3	Chai-1
Chai-1	<2e-16	-
RF2NA	0.0053	1.0000

P value adjustment method: bonferroni

Species: **N_subflava**

Pairwise Wilcoxon test for PAE:

Pairwise comparisons using Wilcoxon rank sum test with continuity correction

data: PAE and Software

	AF3	Chai-1
Chai-1	<2e-16	-
RF2NA	<2e-16	<2e-16

P value adjustment method: bonferroni

Pairwise Wilcoxon test for pLDDT:

Pairwise comparisons using Wilcoxon rank sum test with continuity correction

data: plddt and Software

	AF3	Chai-1
Chai-1	0.00027	-
RF2NA	< 2e-16	< 2e-16

P value adjustment method: bonferroni

```
>
> # Calculate median PAE and plddt values by Software for each Species
> median_values <- merged_df %>%
+   group_by(Species, Software) %>%
+   summarise(
+     median_PAE = median(PAE, na.rm = TRUE),
+     median_plddt = median(plddt, na.rm = TRUE),
+     .groups = "drop"
+   )
>
> # Print median values with species labels
> for (species in unique(median_values$Species)) {
```

```
+   cat("\nSpecies:", species, "\n")
+   print(median_values %>% filter(Species == species))
+ }
```

PAE and pLDDT

```
> # Perform pairwise Wilcoxon test for PAE and plddt with Bonferroni correction for each Species
> results <- merged_df %>%
+   group_by(Species) %>%
+   summarise(
+     pairwise_pa = list(pairwise.wilcox.test(PAE, Software, p.adjust.method = "bonferroni")),
+     pairwise_plddt = list(pairwise.wilcox.test(plddt, Software, p.adjust.method = "bonferroni"))
+   )
>
> # Print results with species labels
> for (i in 1:nrow(results)) {
+   cat("\nSpecies:", results$Species[i], "\n")
+   cat("Pairwise Wilcoxon test for PAE:\n")
+   print(results$pairwise_pa[[i]])
+   cat("Pairwise Wilcoxon test for pLDDT:\n")
+   print(results$pairwise_plddt[[i]])
+ }
```

Species: **B_denitrificans**

```
# A tibble: 3 × 4
  Species         Software  median_PAE  median_plddt
  <chr>           <chr>          <dbl>         <dB>
1 B_denitrificans AF3             4.07          86.9
2 B_denitrificans Chai-1          4.14          87.0
3 B_denitrificans RF2NA           9.90          84.0
```

Species: E_corrodens

A tibble: 3 × 4

	Species	Software	median_PAE	median_plddt
	<chr>	<chr>	<dbl>	<dbl>
1	E_corrodens	AF3	4.01	85.0
2	E_corrodens	Chai-1	2.89	88.1
3	E_corrodens	RF2NA	9.66	82.8

Species: **K_denitrificans**

A tibble: 3 × 4

	Species	Software	median_PAE	median_plddt
	<chr>	<chr>	<dbl>	<dbl>
1	K_denitrificans	AF3	3.51	90.8
2	K_denitrificans	Chai-1	3.52	85.6
3	K_denitrificans	RF2NA	9.48	83.4

Species: **N_cinerea**

A tibble: 3 × 4

	Species	Software	median_PAE	median_plddt
	<chr>	<chr>	<dbl>	<dbl>
1	N_cinerea	AF3	5.34	82.4
2	N_cinerea	Chai-1	3.05	85.4
3	N_cinerea	RF2NA	9.86	83.6

Species: **N_meningitidis**

A tibble: 3 × 4

	Species	Software	median_PAE	median_plddt
	<chr>	<chr>	<dbl>	<dbl>
1	N_meningitidis	AF3	5.73	79.9
2	N_meningitidis	Chai-1	3.30	86.3
3	N_meningitidis	RF2NA	9.52	84.2

Species: **N_mucosa**

A tibble: 3 × 4

	Species	Software	median_PAE	median_plddt
	<chr>	<chr>	<dbl>	<dbl>
1	N_mucosa	AF3	4.09	82.4
2	N_mucosa	Chai-1	4.72	83.4

```
3 N_mucosa RF2NA       9.89        83.7
```

Species**: N_subflava**

```
# A tibble: 3 × 4
  Species    Software      median_PAE    median_plddt
  <chr>      <chr>              <dbl>           <dbl>
1 N_subflava AF3                 4.04            85.9
2 N_subflava Chai-1              3.17            87.6
3 N_subflava RF2NA               9.50            82.0
```
