# Supplementary material for: Two different and robustly modeled DNA binding modes of Competence Protein ComP - systematic modeling with AlphaFold 3, RoseTTAFold2NA, Chai-1 and re-docking in HADDOCK
Source: PLoS One. 2025 May 8;20(5):e0315160. doi: 10.1371/journal.pone.0315160 (PMC12061091; doi:10.1371/journal.pone.0315160)
Supplement: S2 Code Output — (PDF) [file pone.0315160.s011.pdf]

Code Output S2. Wilcoxon rank-sum tests on ipTM and CPPM for each $ComP_{nat}$.

```
### Native vs. Scrambled DUS ###

> ### Fisher's test for ipTM and CPPM differences across Pairing ###
>
> ### ipTM ###
>
> ### AA-king3DUS
>
> # Subset data for 'Native' Pairing
> group_native <- merged_df_filtered$ipTM[merged_df_filtered$DUS == "aa_king3dus"
&
+                               merged_df_filtered$Pairing == "Native"]
>
> # Subset data for 'Scrambled' Pairing
> group_scrambled <- merged_df_filtered$ipTM[merged_df_filtered$DUS ==
"aa_king3dus" &
+                                 merged_df_filtered$Pairing == "Scrambled"]
>
> # Test for normality in the 'Native' group
> shapiro_native <- shapiro.test(group_native)
>
> # Test for normality in the 'Scrambled' group
> shapiro_scrambled <- shapiro.test(group_scrambled)
>
> # Print the results
> # print(shapiro_native)
> # print(shapiro_scrambled)
>
> aa_king3dus_df <- subset(merged_df_filtered, DUS == 'aa_king3dus')
>
> # Perform Wilcoxon rank-sum test (non-parametric)
> wilcox_test_result <- wilcox.test(ipTM ~ Pairing, data = aa_king3dus_df,
+                          exact = FALSE)
>
> # Print the results
> print(wilcox_test_result)
```

```
	Wilcoxon rank sum test with continuity correction

data:  ipTM by Pairing
W = 5395.5, p-value = 0.7319
alternative hypothesis: true location shift is not equal to 0

>
> ### AG-DUS
>
> # Subset data for 'Native' Pairing
> group_native <- merged_df_filtered$ipTM[merged_df_filtered$DUS == "ag_dus" &
+                                 merged_df_filtered$Pairing == "Native"]
>
> # Subset data for 'Scrambled' Pairing
> group_scrambled <- merged_df_filtered$ipTM[merged_df_filtered$DUS == "ag_dus"
&
+                                  merged_df_filtered$Pairing == "Scrambled"]
>
> # Test for normality in the 'Native' group
> shapiro_native <- shapiro.test(group_native)
>
> # Test for normality in the 'Scrambled' group
> shapiro_scrambled <- shapiro.test(group_scrambled)
>
> # Print the results
> # print(shapiro_native)
> # print(shapiro_scrambled)
>
> ag_dus_df <- subset(merged_df_filtered, DUS == 'ag_dus')
>
> # Perform Wilcoxon rank-sum test (non-parametric)
> wilcox_test_result <- wilcox.test(ipTM ~ Pairing, data = ag_dus_df,
+                            exact = FALSE, alternative = "greater")
>
> # Print the results
> print(wilcox_test_result)
```

```
	Wilcoxon rank sum test with continuity correction

data:  ipTM by Pairing
W = 115384, p-value < 2.2e-16
alternative hypothesis: true location shift is greater than 0

>
> ### AG-eikDUS
>
> # Subset data for 'Native' Pairing
> group_native <- merged_df_filtered$ipTM[merged_df_filtered$DUS == "ag_eikdus" &
+                               merged_df_filtered$Pairing == "Native"]
>
> # Subset data for 'Scrambled' Pairing
> group_scrambled <- merged_df_filtered$ipTM[merged_df_filtered$DUS ==
"ag_eikdus" &
+                                 merged_df_filtered$Pairing == "Scrambled"]
>
> # Test for normality in the 'Native' group
> shapiro_native <- shapiro.test(group_native)
>
> # Test for normality in the 'Scrambled' group
> shapiro_scrambled <- shapiro.test(group_scrambled)
>
> # Print the results
> # print(shapiro_native)
> # print(shapiro_scrambled)
>
> ag_eikdus_df <- subset(merged_df_filtered, DUS == 'ag_eikdus')
>
> # Perform Wilcoxon rank-sum test (non-parametric)
> wilcox_test_result <- wilcox.test(ipTM ~ Pairing, data = ag_eikdus_df,
+                          exact = FALSE, alternative = "greater")
>
> # Print the results
> print(wilcox_test_result)
```

```
	Wilcoxon rank sum test with continuity correction

data:  ipTM by Pairing
W = 8867.5, p-value < 2.2e-16
alternative hypothesis: true location shift is greater than 0

>
> ### AG-mucDUS
>
> # Subset data for 'Native' Pairing
> group_native <- merged_df_filtered$ipTM[merged_df_filtered$DUS == "ag_mucdus"
&
+                                merged_df_filtered$Pairing == "Native"]
>
> # Subset data for 'Scrambled' Pairing
> group_scrambled <- merged_df_filtered$ipTM[merged_df_filtered$DUS ==
"ag_mucdus" &
+                                 merged_df_filtered$Pairing == "Scrambled"]
>
> # Test for normality in the 'Native' group
> shapiro_native <- shapiro.test(group_native)
>
> # Test for normality in the 'Scrambled' group
> shapiro_scrambled <- shapiro.test(group_scrambled)
>
> # Print the results
> # print(shapiro_native)
> # print(shapiro_scrambled)
>
> ag_mucdus_df <- subset(merged_df_filtered, DUS == 'ag_mucdus')
>
> # Perform Wilcoxon rank-sum test (non-parametric)
> wilcox_test_result <- wilcox.test(ipTM ~ Pairing, data = ag_mucdus_df,
+                           exact = FALSE, alternative = "greater")
>
> # Print the results
```

```
> print(wilcox_test_result)

	Wilcoxon rank sum test with continuity correction

data:  ipTM by Pairing
W = 9364, p-value < 2.2e-16
alternative hypothesis: true location shift is greater than 0

>
> ### AT-DUS
>
> # Subset data for 'Native' Pairing
> group_native <- merged_df_filtered$ipTM[merged_df_filtered$DUS == "at_dus" &
+                                merged_df_filtered$Pairing == "Native"]
>
> # Subset data for 'Scrambled' Pairing
> group_scrambled <- merged_df_filtered$ipTM[merged_df_filtered$DUS == "at_dus"
&
+                                 merged_df_filtered$Pairing == "Scrambled"]
>
> # Test for normality in the 'Native' group
> shapiro_native <- shapiro.test(group_native)
>
> # Test for normality in the 'Scrambled' group
> shapiro_scrambled <- shapiro.test(group_scrambled)
>
> # Print the results
> # print(shapiro_native)
> # print(shapiro_scrambled)
>
> at_dus_df <- subset(merged_df_filtered, DUS == 'at_dus')
>
> # Perform Wilcoxon rank-sum test (non-parametric)
> wilcox_test_result <- wilcox.test(ipTM ~ Pairing, data = at_dus_df,
+                           exact = FALSE, alternative = "less")
>
> # Print the results
```

```
> print(wilcox_test_result)

	Wilcoxon rank sum test with continuity correction

data:  ipTM by Pairing
W = 7496, p-value < 2.2e-16
alternative hypothesis: true location shift is less than 0

>
> ### CPPM ###
>
> ### AA-king3DUS
>
> # Subset data for 'Native' Pairing
> group_native <- merged_df_filtered$Chain_pair_pae_min[merged_df_filtered$DUS
== "aa_king3dus" &
+                                            merged_df_filtered$Pairing == "Native"]
>
> # Subset data for 'Scrambled' Pairing
> group_scrambled <-
merged_df_filtered$Chain_pair_pae_min[merged_df_filtered$DUS == "aa_king3dus"
&
+                                              merged_df_filtered$Pairing == "Scrambled"]
>
> # Test for normality in the 'Native' group
> shapiro_native <- shapiro.test(group_native)
>
> # Test for normality in the 'Scrambled' group
> shapiro_scrambled <- shapiro.test(group_scrambled)
>
> # Print the results
> # print(shapiro_native)
> # print(shapiro_scrambled)
>
> aa_king3dus_df <- subset(merged_df_filtered, DUS == 'aa_king3dus')
>
> # Perform Wilcoxon rank-sum test (non-parametric)
```

```
> wilcox_test_result <- wilcox.test(Chain_pair_pae_min ~ Pairing, data =
aa_king3dus_df,
+                                   exact = FALSE)
>
> # Print the results
> print(wilcox_test_result)

	Wilcoxon rank sum test with continuity correction

data:  Chain_pair_pae_min by Pairing
W = 5884.5, p-value = 0.1353
alternative hypothesis: true location shift is not equal to 0

>
> ### AG-DUS
>
> # Subset data for 'Native' Pairing
> group_native <- merged_df_filtered$Chain_pair_pae_min[merged_df_filtered$DUS
== "ag_dus" &
+                                                       merged_df_filtered$Pairing == "Native"]
>
> # Subset data for 'Scrambled' Pairing
> group_scrambled <-
merged_df_filtered$Chain_pair_pae_min[merged_df_filtered$DUS == "ag_dus" &
+                                                          merged_df_filtered$Pairing == "Scrambled"]
>
> # Test for normality in the 'Native' group
> shapiro_native <- shapiro.test(group_native)
>
> # Test for normality in the 'Scrambled' group
> shapiro_scrambled <- shapiro.test(group_scrambled)
>
> # Print the results
> # print(shapiro_native)
> # print(shapiro_scrambled)
>
> ag_dus_df <- subset(merged_df_filtered, DUS == 'ag_dus')
```

```
>
> # Perform Wilcoxon rank-sum test (non-parametric)
> wilcox_test_result <- wilcox.test(Chain_pair_pae_min ~ Pairing, data = ag_dus_df,
+                                   exact = FALSE, alternative = "less")
>
> # Print the results
> print(wilcox_test_result)

        Wilcoxon rank sum test with continuity correction

data:  Chain_pair_pae_min by Pairing
W = 18604, p-value < 2.2e-16
alternative hypothesis: true location shift is less than 0

>
```

> ### **AG-eikDUS**

```
>
> # Subset data for 'Native' Pairing
> group_native <- merged_df_filtered$Chain_pair_pae_min[merged_df_filtered$DUS
== "ag_eikdus" &
+                                                     merged_df_filtered$Pairing == "Native"]
>
> # Subset data for 'Scrambled' Pairing
> group_scrambled <-
merged_df_filtered$Chain_pair_pae_min[merged_df_filtered$DUS == "ag_eikdus" &
+                                                        merged_df_filtered$Pairing == "Scrambled"]
>
> # Test for normality in the 'Native' group
> shapiro_native <- shapiro.test(group_native)
>
> # Test for normality in the 'Scrambled' group
> shapiro_scrambled <- shapiro.test(group_scrambled)
>
> # Print the results
> # print(shapiro_native)
> # print(shapiro_scrambled)
>
```

```
> ag_eikdus_df <- subset(merged_df_filtered, DUS == 'ag_eikdus')
>
> # Perform Wilcoxon rank-sum test (non-parametric)
> wilcox_test_result <- wilcox.test(Chain_pair_pae_min ~ Pairing, data = ag_eikdus_df,
+                               exact = FALSE, alternative = "less")
>
> # Print the results
> print(wilcox_test_result)

	Wilcoxon rank sum test with continuity correction

data:  Chain_pair_pae_min by Pairing
W = 1840, p-value = 5.809e-15
alternative hypothesis: true location shift is less than 0

>
```

> ### **AG-mucDUS**

```
>
> # Subset data for 'Native' Pairing
> group_native <- merged_df_filtered$Chain_pair_pae_min[merged_df_filtered$DUS
== "ag_mucdus" &
+                                         merged_df_filtered$Pairing == "Native"]
>
> # Subset data for 'Scrambled' Pairing
> group_scrambled <-
merged_df_filtered$Chain_pair_pae_min[merged_df_filtered$DUS == "ag_mucdus" &
+                                         merged_df_filtered$Pairing == "Scrambled"]
>
> # Test for normality in the 'Native' group
> shapiro_native <- shapiro.test(group_native)
>
> # Test for normality in the 'Scrambled' group
> shapiro_scrambled <- shapiro.test(group_scrambled)
>
> # Print the results
> # print(shapiro_native)
> # print(shapiro_scrambled)
```

```
>
> ag_mucdus_df <- subset(merged_df_filtered, DUS == 'ag_mucdus')
>
> # Perform Wilcoxon rank-sum test (non-parametric)
> wilcox_test_result <- wilcox.test(Chain_pair_pae_min ~ Pairing, data =
ag_mucdus_df,
+                         exact = FALSE, alternative = "less")
>
> # Print the results
> print(wilcox_test_result)

	Wilcoxon rank sum test with continuity correction

data:  Chain_pair_pae_min by Pairing
W = 1200, p-value < 2.2e-16
alternative hypothesis: true location shift is less than 0

>
> ### AT-DUS
>
> # Subset data for 'Native' Pairing
> group_native <- merged_df_filtered$Chain_pair_pae_min[merged_df_filtered$DUS
== "at_dus" &
+                                   merged_df_filtered$Pairing == "Native"]
>
> # Subset data for 'Scrambled' Pairing
> group_scrambled <-
merged_df_filtered$Chain_pair_pae_min[merged_df_filtered$DUS == "at_dus" &
+                                   merged_df_filtered$Pairing == "Scrambled"]
>
> # Test for normality in the 'Native' group
> shapiro_native <- shapiro.test(group_native)
>
> # Test for normality in the 'Scrambled' group
> shapiro_scrambled <- shapiro.test(group_scrambled)
>
> # Print the results
```

```
> # print(shapiro_native)
> # print(shapiro_scrambled)
>
> at_dus_df <- subset(merged_df_filtered, DUS == 'at_dus')
>
> # Perform Wilcoxon rank-sum test (non-parametric)
> wilcox_test_result <- wilcox.test(Chain_pair_pae_min ~ Pairing, data = at_dus_df,
+                               exact = FALSE, alternative = "greater")
>
> # Print the results
> print(wilcox_test_result)

	Wilcoxon rank sum test with continuity correction

data:  Chain_pair_pae_min by Pairing
W = 32456, p-value < 2.2e-16
alternative hypothesis: true location shift is greater than 0
```
