# Supplementary material for: Two different and robustly modeled DNA binding modes of Competence Protein ComP - systematic modeling with AlphaFold 3, RoseTTAFold2NA, Chai-1 and re-docking in HADDOCK
Source: PLoS One. 2025 May 8;20(5):e0315160. doi: 10.1371/journal.pone.0315160 (PMC12061091; doi:10.1371/journal.pone.0315160)
Supplement: S3 Code Output — (PDF) [file pone.0315160.s012.pdf]

Code Output S3 Wilcoxon rank-sum test on DockQ scores for the internal platform consistency check.

```
> # Display results
> test_af3_chai

	Wilcoxon rank sum test with continuity correction

data:  af3_df$DockQ and chai_df$DockQ
W = 1.3061e+10, p-value < 2.2e-16
alternative hypothesis: true location shift is not equal to 0

> test_af3_rf2na

	Wilcoxon rank sum test with continuity correction

data:  af3_df$DockQ and rf2na_df$DockQ
W = 432365430, p-value < 2.2e-16
alternative hypothesis: true location shift is not equal to 0

> test_chai_rf2na

	Wilcoxon rank sum test with continuity correction

data:  chai_df$DockQ and rf2na_df$DockQ
W = 3699847036, p-value < 2.2e-16
alternative hypothesis: true location shift is not equal to 0

>
> median(af3_df$DockQ)
[1] 0.841
> median(chai_df$DockQ)
[1] 0.68
> median(rf2na_df$DockQ)
[1] 0.617
```
